# Ultrasensitive Optical Chemosensor for Cu(II) Detection

**DOI:** 10.1155/2019/7381046

**Published:** 2019-03-26

**Authors:** Sayed M. Saleh, Reham Ali, Fahad Alminderej, Ibrahim A. I. Ali

**Affiliations:** ^1^Chemistry Department, Science College, Qassim University, Buraidah, Saudi Arabia; ^2^Chemistry Branch, Department of Science and Mathematics, Faculty of Petroleum and Mining Engineering, Suez University, Suez, Egypt; ^3^Chemistry Department, Science College, Suez University, Suez, Egypt; ^4^Chemistry Department, Science College, Suez Canal University, Ismailia, Egypt

## Abstract

Herein, the main objective of this research is to design and synthesize a novel optical chemosensor, 2,6-Bis(4-dimethylaminophenyl)-4-(dicyanomethylene)-cyclohexane-1,1-dicarbo-nitrile (BDC), for detection of one of the most significant metal ions Cu(II). This novel fluorescent chemosensor exhibits unique optical properties with large Stokes shift (about 170 nm) approximately. The fluorescence and UV–vis absorption performance among the BDC probe and Cu(II) ions were examined in 1:9 (v/v) methanol–HEPES buffer (pH = 7.2) solution. Also, BDC displays high selectivity for Cu(II) concerning other cations. Moreover, this probe provides high selectivity and sensitivity based on their fluorescence properties and recognition abilities within a detection limit of the Cu(II) contents (LOD 2.3 x 10^−7^ M). The suggested mechanism of BDC sensor is attributed to the chelation process with Cu(II), to establish a 1:1 metal-ligand ratio complex with a binding constant (K_bind_ = 7.16 x 10^4^ M^−1^). The detection process is accompanied by quenching the main emission peak of the BDC at 571 nm. All the experimental data were collected to investigate the effects of several important parameters such as reversibility and the concentration limits. Besides, we study the interference of various metal ions on selectivity and detection capacity of this significant Cu (II) ion. This novel chemosensor shows ultrasensitive, fast tracing of Cu(II) in the physiological pH range (pH 7.2) and therefore may propose a novel promising method for the investigation of the biological functions of Cu(II) in living cells.

## 1. Introduction

As one of the most essential metal ions in humans, copper ions play a vital role in several biological processes involving generation of cellular energy, transferring and activation of oxygen molecules, and transduction of signals [1]. According to the limitations of U. S. Environmental Protection Agency (EPA), the maximum Cu(II) ion content in drinking water is 1.3 mg/L [2–4]. Thus, in case of increasing Cu(II) content, these ions bind to the active sites of some particular enzymes and exchange the nutrient minerals [5–8]. During this process, the metal-enzyme interaction alters the functions of many enzymes [9, 10], which causes disturbance of the same biological systems. Therefore, the enzymatic activity of these enzymes decreases significantly to about 5 % of their natural activity. Additionally, copper ions may cause many health problems. The structure of many tissues changes during absorption process of these toxic heavy metal ions, followed by replacing other substituents. These tissues (e.g., the arteries, joints, bones, and muscles) are affected strongly with this undesired interaction and cause health weakness and a series of dangerous diseases [11–14]. Also, the deposition of copper ions in many sites causes irritation and other lethal effects [15]. The metal ions absorption also distorts the DNA replication and synthesis of many bioprocesses. This process causes critical alteration of the body chemistry-based RNA and DNA [16, 17].

The significance of sensing and detection of heavy metals in aquatic samples is unquestionable [18, 19]. The tracing of the heavy metal ions in the environment (biological sample or wastewater) has several significant limitations such as the low detection limit, the sensitivity of investigated techniques, and the interference with system components. In conventional methods, the detection limit of the utilized techniques is essential. Several techniques were applied for the detection of heavy metal ions such as flame atomic absorption spectrometry (FAAS), inductively coupled plasma (ICP), etc. [20, 21]. However, these techniques require special samples preparation and perusal procedures, expensive cost, professional handling. Furthermore, they are not portable instruments and need a stationary lab for the detection process.

The optical chemosensors exhibit fluorescence or color changes upon the detection process of the heavy metal ions. Most recently researches for detection Cu(II) ion based fluorescence chemosensors are considered as molecular self-absorption because they suffer from small Stokes shift. [22–24]. Large Stokes shift limits reabsorption of emitted light and scattering, which provides cellular imaging with high signal-to-noise ratio [25, 26]. There are several advantages which involve the use of optical sensors such as response rate and results precision [27–33]. However, the usage of fluorescence-based optical chemical sensing technique is not commonly employed. Concerning other spectroscopic tools, this method provides a very sensitive and simple readout. Moreover, this sensing technique offers real-time recognition for the analyte, which improves the chemical analyses field. Also, it has significant applications in many biological and medical issues, such as blood analysis and cell imaging [34–36]. Interestingly, the net results of the chemosensors will offer a complete view about the sensing mechanism and limit of detection of the heavy metal content. Eventually, this will enable inhibiting the usage of contaminated water and food that polluted with heavy metal ions.

Many chemosensors were developed, designed, and synthesized for determination copper ions with high proficiency, precision, and measurability [37]. These chemosensors have been developed based on the significant organic probe such as some traditional organic dyes [38]. Besides, the appropriate mechanism of these organic probes depends on various optical sensing mechanisms such as ratiometric fluorescence mechanism [39–41] and fluorescence resonance energy transfer FRET [42]. These specified sensing probes dyes cover a wide range of emission spectrum from UV to near IR [43]. One of the most important chemosensors for sensing copper ions has been developed based on Turn-Off mechanism. This mechanism provides the quenching of the fluorescent organic probe due to the chelation process with copper ions. Numerous types of signaling mechanisms have been reported and applied for optical tracing of several cations, involving intra-molecular charge transfer (ICT) [44], photo-induced energy transfer (PET) [45, 46], charge transfer based metal-ligand interactions (CTML) [47], excimer/exciplex formation (EEF) [48], and excited state intra/inter-molecular proton transfer (ESPIT) [49]. The design and synthesis of the chemosensors depend mainly on the mechanism type which controls the interaction between the specified metal ions and the sensing molecules. The paramagnetic nature of the copper ions provides the quenching property of the intrinsic fluorescence of the chemosensors [50]; the majority of researches described that copper ions chemosensors had exhibited quenched emission during binding interaction [51]. The main target of this work is to design and synthesize new and highly sensitive optical chemosensors BDC for detection of copper metal ions. This chemosensor can determine the copper ion contents within a concentration limit of 2.3 x 10^−7^ M (LOD). Further, this novel sensor has the prospect to show fast responsivity, easy reversibility, and reproducibility for copper metal ions detection.

## 2. Materials and Methods

### 2.1. Instruments and Materials

NMR spectra of 2,6-Bis(4-dimethylaminophenyl)-4-(dicyanomethylene)-cyclohexane-1,1-dicarbonitrile were attained at 25°C on a Bruker Avance DRX200 (200 MHz-1H) or Bruker Avance DRX500 (125 MHz for ^13^C) spectrometer with calibration to the residual proton solvent signal of DMSO-d_6_. Melting point was detected on a Stuart-SM/P3 instrument in a preserved capillary. The UV–vis spectra were obtained using a 1-cm quartz cell utilizing (Evolution™_200-series/UV-Visible) spectrophotometer. Fluorescence spectra were determined on a (JASCOFP6300-spectrofluorometer) using a 1 cm quartz cell for the emission and excitation spectral measurements. Chemicals were obtained from Sigma–Aldrich and used without further purification: dimethylamino benzaldehyde, malononitrile, sodium acetate, and metal nitrates.

### 2.2. Synthesis of BDC

**Figure 1:**
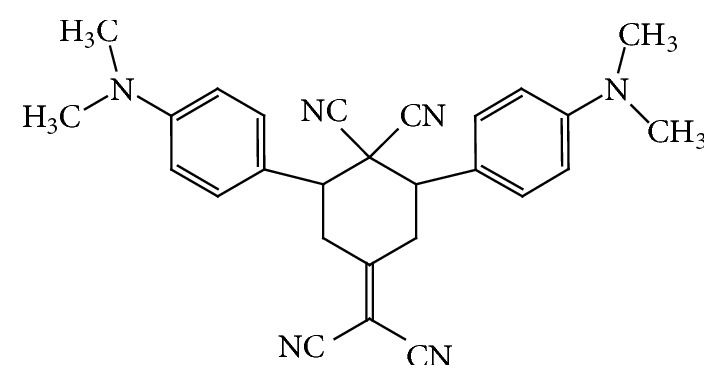
Chemical structure of 2,6-Bis(4-dimethylaminophenyl)-4-(dicyanomethylene)-cyclohex-ane-1,1-dicarbonitrile.

The chemosensor probe was synthesized according to the recent literature [52]. Typically, A solution of dimethylamino benzaldehyde (20.0 mmol), malononitrile (15.0 mmol), and sodium acetate (2.0 mg, 0.02 mmol) in 20 acetone were stirred at 20°C for 5 h. The solid phase was filtered and washed with cold acetone to yield compound 79 %; the chemical structure of the yield BDC is shown in Figure 1. M_p_=234°C; ^1^HNMR (300 MHz, CDCl_3_):*δ* = 3.00 (2s, 12 H, 4 CH_3_), 3.30-4.10 (m, 4 H, 2 CH_2_), 4.25-5.51 m, 2 H, 2 CH, 6.58-6.73 (m, 4 H, Ar-H), 7.85-8.03 (m, 4 H, Ar-H); FTIR *υ*_max_ 2240, 1616, 1544, 1440, 762. Elemental Analyses calculated for C_27_H_26_N_6_: C, 74.65; H, 5.99; N, 19.35 and Found: C, 74.49; H, 5.89; N, 19.28.

### 2.3. Optical Measurements.

Different nitrate salt solutions of Cu^2+^, Ni^2+^, Hg^2+^, Ba^2+^, Mg^2+^, Ag^+^, Fe^2+^, K^+^, Al^3+^, Mn^2+^, Pb^2+^, Na^+^, Sr^2+^, Co^2+^, Zn^2+^, Cd^2+^, Cr^3+^and Fe^3+^ with 1x10^−3^ M concentration were prepared for all metal cations, and a stock solution of 1x10^−3^ M BDC probe was prepared in methanol. The investigated solutions were prepared by mixing appropriate volume of the salt ions solution with a particular aliquot of probe stock solution and diluting the net solution. The absorption spectra (UV−vis) of the chemosensor probe and the formed metal-probe complexes were detected in methanol-HEPES buffer mixture of (1:9, v/v) with pH = 7.2. The fluorescence measurements were investigated for the free probe in absence of any metal ions and the metal-chemosensor complexes under the same conditions. In a photometric titration, the net volume of the free sensor probe and salt ions could be fixed at 2.2 mL because the volume of salt ions can be neglected concerning the volume of the free sensing probe. For all fluorescence spectra, measurements for both emission and excitation slit widths were 5 nm.

### 2.4. Study the Binding Complex Stoichiometry and Reversibility

To study the stoichiometry of the Cu(II) metal-BDC complex, Job's method based on fluorescence measurements was utilized. Preserving the total of the initial concentration of Cu(II) ion and BDC free ligand at 10 *μ*M, and varying the molar ratio of Cu(II) within range of 0.1, 0.2, 0.3, 0.4, 0.5, 0.6 0.7, 0.8, 0.9. The fluorescence of BDC in the absence (F_0_) and presence F of Cu(II) ions were detected, respectively. A plot of (F_0_ - F) versus different molar ratios of Cu(II) ions was drawn to investigate the stoichiometry of metal complex formation. For the recovery and reversibility experiments, a 2.2 mL of mixture solution containing Cu(II) ions and BDC ligand molecules with a molar ratio of (1:1) was prepared in methanol-HEPES buffer mixture of (1:9, v/v) pH = 7.2 solution. The resulting solution has 10 *μ*M Cu(II)-BDC complex. 5 *μ*L aliquots of 1 x 10^−3^ M EDTA solution were added gradually to the metal-complex solution in order to investigate the reversibility of the optical chemical sensor.

### 2.5. Quantum Yield

The fluorescence quantum yields of BDC free ligand and Cu(II) metal complex were calculated utilizing the optical dilute route based on standard reference solution of quinine sulfate in 0.05 M sulfuric acid. This standard solution provides 55 % quantum yield at 298°K [53]. All sample solutions were measured in quartz cell of path length 1 cm. This cell provides absorbance value below 0.1 for all wavelength range of excitation and emission to consistently illumine through the sample and to cancel the influence of inner-filter effect.

## 3. Results and Discussion

### 3.1. Optical Measurements of BDC

**Figure 2:**
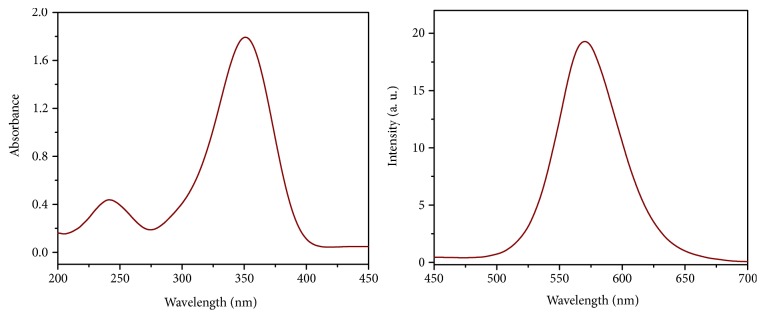
The optical properties include UV-Vis and emission spectra of the BDC.

The synthesized 2,6-Bis(4-(dimethylamino)phenyl)-4-(dicyanomethylene)cyclohexane-1,1-dicarbonitrile shows distinctive optical properties; these properties were measured in methanol-HEPES buffer mixture of (1:9, v/v) with pH = 7.2 (see Figure 2). Under excitation with 401 nm, the free probe exhibits a characterized peak centered at 571 nm. On the other hand, the absorption spectra indicate presence of two peaks (242 and 351 nm, respectively).

**Figure 3:**
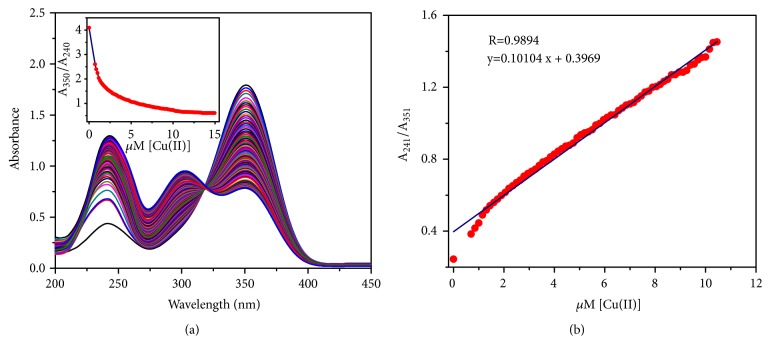
(a) Absorption spectral alterations of chemosensor BDC upon gradual addition of (0–2 equiv.) Cu(II); (b) the linear relationship of the absorbance ratio at 351 and 241 nm (A_241_/A_351_) versus [Cu(II)].

The fluorescence response of the BDC towards the copper ions was detected using absorption and emission techniques. Upon adding up Cu(II) ions to the plain BDC solution, a new absorption band generated at 301 nm, besides the two main absorption peaks of the free BDC probe, which could be attributed to *π*-*π∗* and n-*π∗* transitions [54]. The absorption of the new and the 242 nm peaks is enhanced with further addition of copper ions. On the contrary, the 351 absorption peak starts to quench upon addition of copper ions to the BDC solution. The synchronous action of the three absorption peaks proceeds with appearance of an isosbestic point at 318 nm (Figure 3(a)). Interestingly, unique behavior of BDC UV-vis spectra confirms the chelation process between Cu(II) metal and the nitrile groups of the sensitive chemosensor [55].

Memorable, a significant linear relationship exhibits the relation between absorbance ratio at 241 and 351 nm of the chemosensor probe and the Cu(II) concentration within a range of 0 to 1.0 x10^−5^ M (Figure 3(b)). Further, with increasing Cu(II) concentration up to a molar ratio of BDC to metal ions of 1:1, the plot shows linearly dependent coefficient: R^2^= 0.9894, and the absorption ratios becomes steady at a higher molar ratio. Thus, the ratiometric detection process exhibits a 1:1 Cu(II) binding stoichiometry of BDC. Considerably, the limit of detection (LOD) of BDC chemosensor probe was determined to be 2.3 x 10^−7^ M, using the calculation formula LOD = 3*σ*/k, where k and *σ* give the liner curve slope and standard deviation, respectively [56]. Furthermore, BDC has four cyanide groups which have significantly observed a considerable inorganic ligand. There are some significant explanations for this assessment, such as the high similarity to halides, its stability as an aqueous anion, and its capability to form salts with numerous metals. However, there are possibly better explanations to classify metal−cyano complexes as organometallics [57, 58].

**Figure 4:**
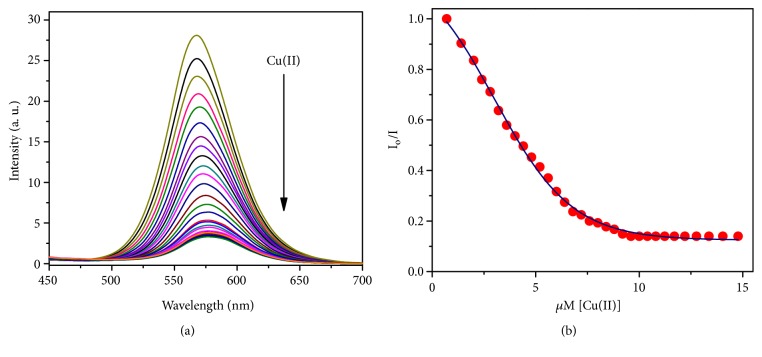
Fluorescence emission spectra of BDC 10 *μ*M in 1:9 Methanol HEPES buffer solution (pH = 7.2) during gradually addition of Cu(II) ions within range of 0-15 *μ*M, under excitation with 401 nm.

Concurrently, BDC chemosensor probe has an orange fluorescence with the main peak appeared at 571 nm and under excitation with 401 nm. The optical properties of BDC show that there is no overlap between the absorption and fluorescence spectra; and large Stokes shift (Δ*λ* = 170 nm) was recorded. Most organic probes that are used for Cu(II) ion detection generally exhibit small Stokes shifts, which would increase cross-talk between absorbed and emitted photons and scattering [59]. This leads to undesired back-ground interferences which requires the using of other fluorophore to introduce energy transfer between the two flourophores to increase Stokes shifts. Also, the large Stokes shift minimizes self-quenching which provides a significant feature in practical applications. When the Cu(II) was gradually added to BDC, a substantial quenching of the fluorescence intensity of the main peak at 571 nm was observed, as shown in Figure 4(a). Upon addition of Cu(II) ion to the free ligand solution within a concentration range of 1:1 (0.64 mg/L to 4.3 mg/L, respectively), the main emission peak reaches minimum intensity value. After adding more than 1 equivalent of Cu(II) ions, the fluorescence intensity became a minimum and the fluorescence titration exhibits nonlinear curve fitting of the fluorescence emission titration and provides a 1:1 complex ratio between the BDC chemosensor probe and Cu(II) ions, which was in conformity with absorption titration data (see Figure 4(b)).

**Figure 5:**
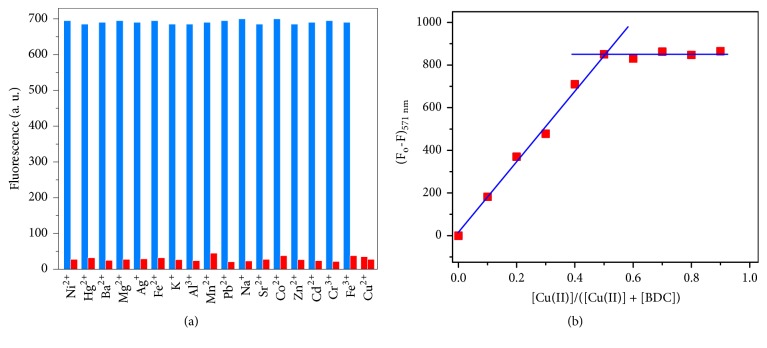
(a) Emission intensity of BDC chemosensor probe (I) in the presence of different cations (blue bar) and (II) in the presence of same cations besides Cu(II) ions (red bar). BDC: Cu(II): M(II) is equal to 1:1:1 (*λ*_ex_ = 401 nm); (b) Job's plot for detection the stoichiometric chelation of BDC with Cu(II) ion in 1:9 (v/v) methanol-HEPES buffer solution (pH = 7.2); fluorescence intensities versus mole fraction of Cu(II) ion were plotted.

Upon addition of various metal ions with a concentration of 10 *μ*M, the chelation of each metal ion to 10 *μ*M BDC chemosensor probe was studied separately in 1:9 methanol-HEPES buffer solution (pH=7.2) utilizing fluorescence spectroscopy. There were minimal changes in the emission intensities of the BDC probe. As shown in Figure 5(a), the effect of each metal ion on the sensitivity of BDC in presence of Cu(II) ions was investigated; all the investigated interfering salt ions have no noticeable interference within the chemical sensing of Cu(II) ions based BDC sensor. Thus, the chemosensor could be utilized as a selective sensor for Cu(II). The stoichiometric ratio (BDC: Cu(II) complex) was determined using Job's plot based fluorescence analysis [60, 61]. Job's plot (see Figure 5(b)) was performed by fluctuating the concentration of copper metal ions within a range up to 10 *μ*M. The highest quenching value of the fluorescence intensity was detected when the molar ratio of Cu(II) was approximately 0.5, which proved that BDC and Cu(II) ion gave a 1:1 complex. Also, this is in a good agreement with the Absorption titration.

### 3.2. Determination the Binding Constant of the Formed Metal Complex

The binding constant **K**_**bind**_ of Cu(II) complex involving BDC organic probe was obtained by utilizing Ryan and Weber equation model based on fluorescence quenching [62, 63]. This equation states that the metal ion binds at congruent and active sites or organic ligands and only with molar ratio of (1:1) metal/ligand complex is composed. The binding constant of the formed complex (ML) of metal cation (M) and ligand molecule (L) could be depicted at definite pH and constant ionic strength using the following formula:

(1) $$\begin{array}{c} K_{m}=\frac{\left[ML\right]}{\left[M\right]}\times \left[\;L\;\right] \end{array}$$

where [M] gives the molar concentration of free Cu(II); [L] indicates the molar concentration of free (BDC) ligand; [ML] is the molar concentration of the formed ML [(Cu(II)-BDC)].Further, the mass Cu(II) and BDC ligand are expressed in the following:

(2) CM=M+ML

(3) CL=L+ML

where C_M_ and C_L_ are the stoichiometric concentrations of Cu(II) ions and BDC. The fraction of ligand that bound to metal ions (v) could be given regarding the binding constant and the free concentration of metal ion using (1) and (3).

(4) $$\begin{array}{c} v=\frac{\left[ML\right]}{C_{L}}=\frac{K_{M}\left[M\right]}{1+k\left[M\right]} \end{array}$$

The ligand fraction could be exhibited using (2) and (4):

(5) $$\begin{array}{c} v=\frac{K_{M}\left(C_{M}-vC_{L}\right)}{1+K_{M}\left(C_{M}-vC_{L}\right)} \end{array}$$

And from (5), the binding constant of the formed metal complex could be calculated as follows:

(6) $$\begin{array}{c} K_{M}=\frac{v}{\left(C_{M}-vC_{L}\right)\left(1-v\right)} \end{array}$$

Weber et al. [62] studied the fluorescence intensity (I) that diverges linearly with the fraction of the total ligand bound giving the next equation:

(7) $$\begin{array}{c} v=\frac{I_{o}-I}{I_{o}-I_{ML}} \end{array}$$

where I_o_ is the emission intensity of the free ligand and I_ML_ is the minimum emission intensity of the ligand after complexation with metal ions at which the emission intensity does not alter with further metal ion addition.

The metal complex between the Cu(II) and BDC optical probe was formed, and the chelation process was accompanied by fluorescence quenching. Using Rayan and Weber [62] equation the binding constant was calculated to be (K_bind_ = 7.16 x 10^4^ M^−1^). Also, the logarithm of the binding constant of the formed complex (log⁡K_bind_) of Cu(II) ions bound to the BDC ligand is calculated to be 4.8. This could be attributed to the interaction between Cu(II) ions and nitrile groups of BDC and form a stable metal complex [55].

### 3.3. Reversibility of the Optical Chemosensor

Ethelenediaminetetraacetate (EDTA), immensely robust chelating agent for Cu(II) ions, was used for studying the reversibility of the Cu-BDC reaction. 5 *μ*L aliquots of 1 x 10^−3^ M EDTA solution were added gradually to a 2.2 mL solution including Cu-(BDC) complex with a molar ratio of (1:1) metal to ligand with a concentration of (10 *μ*M) in methanol-HEPES buffer mixture of (1:9, v/v) pH = 7.2. As a result of EDTA addition, their molecules start to exchange BDC molecules, and a new complex of Cu-EDTA forms instantaneously. Interestingly, the exchange chelation process was accompanied by fluorescence enhancement due to the liberated chemical probe BDC. Thus, the increasing of the fluorescence intensity was observed until they attain 88% of their intensity of the free form.

### 3.4. Quantum Yield

The estimated values of the quantum yield were introduced for both metal complex Cu-BDC and free ligand to be 0.156 and 0.295, respectively. The quinine sulfate [53] was utilized as standard and reference probe to calculate the estimated values of quantum yields. A solution of quinine sulfate probe, 55 % quantum yield, was prepared in sulfuric acid. The limitation error for the quantum yield was roughly stated to be ± 6 %.

(8) $$\begin{array}{c} Q_{x}=Q_{R}\frac{A_{R.}I_{S}.{ɳ}_{S}^{2}}{A_{S}.I_{R}.{ɳ}_{R}^{2}} \end{array}$$

where R and S point to the BDC and complex referencing solutions; *η* is a refractive index at 298°K; I is the integrated area under emission band; A is the maximum absorption band of all cases.

## 4. Conclusion

In brief, an ultrasensitive and novel turn-off optical chemosensor probe BDC was designed and synthesized for detection the Cu(II) ions based on chelation process even without any interference with several metal ions including Cu^2+^, Ni^2+^, Hg^2+^, Ba^2+^, Mg^2+^, Ag^+^, Fe^2+^, K^+^, Al^3+^, Mn^2+^, Pb^2+^, Na^+^, Sr^2+^, Co^2+^, Zn^2+^, Cd^2+^, Cr^3+^, and Fe^3+^. The colorimetric and absorption ratiometric BDC probe exhibited large Stokes shift 170 nm; the present chemosensor offers a new concept for design optical probe with significant alterations in fluorescence. The BDC could detect Cu(II) ions very easily with precious selectivity and distinctive sensitivity and provided a limit of detection (LOD) 2.3 x 10^−7^ M. An excellent linear relationship between the copper ions and BDC in micro-concentrations was displayed and could afford to utilize in quantitative determination of Cu(II) ions. The sensing mechanism of Cu(II) and BDC was attributed to the complexation between two cyano groups and Cu(II). Ryan and Weber equation was used to determine the binding constant of the Cu(II)-BDC metal complex (K_bind_ = 7.16 x 10^4^ M^−1^). Lastly, the BDC sensing probe shows that unique properties including low detection limit, high complex stability, and the interferences from different metal ions are negligible. Thus, BDC can be provided as a novel, highly selective optical sensor for Cu(II) in bioapplications.

## Data Availability

All the significant data were included in the manuscript.
